# miR-125a-5p upregulation suppresses the proliferation and induces the cell apoptosis of lung adenocarcinoma by targeting NEDD9

**DOI:** 10.3892/or.2022.8466

**Published:** 2022-12-19

**Authors:** Hong Zheng, Jianbo Wu, Jiachen Shi, Chunya Lu, Yuanyuan Wang, Qianqian Sun, Guojun Zhang, Guoqiang Zhao

Oncol Rep 38: 1790–1796, 2017; 10.3892/or.2017.5812

Following the publication of this paper, it was drawn to the Editors’ attention by a concerned reader that certain of the Transwell invasion assay data panels shown in Fig. 3A were strikingly similar to data appearing in different form in other articles written by different authors, but which had already been published elsewhere prior to this paper's submission to *Oncology Reports.* In view of the fact some of these data had already apparently been published previously, the Editor of *Oncology Reports* has decided that this paper should be retracted from the Journal. The authors were asked for an explanation to account for these concerns, but the Editorial Office did not receive a reply. The Editor apologizes to the readership for any inconvenience caused.

